# Quality and Privacy Policy Compliance of Mental Health Care Apps in China: Cross-Sectional Evaluation Study

**DOI:** 10.2196/66762

**Published:** 2025-07-03

**Authors:** Xinying Lin, Xingxing Wu, Ziping Zhu, Danting Chen, Hong Li, Rong Lin

**Affiliations:** 1 The School of Nursing Fujian Medical University Fuzhou China

**Keywords:** mobile apps, mental health, mental health care, content analysis, privacy policy

## Abstract

**Background:**

The global prevalence of mental health issues such as depression and anxiety is escalating, yet access to high-quality care remains severely limited. Although mental health care apps can enhance outcomes, their handling of highly sensitive personal data poses significant privacy risks.

**Objective:**

This study systematically investigated Chinese mental health care apps, assessing their quality and privacy policy compliance through an integrated framework of the Information Security Technology–Personal Information Security Specification (GB/*t* 35273-2020) and the Mobile App Rating Scale (MARS).

**Methods:**

A comprehensive search on the Chinese iOS and Android platforms identified apps for evaluation by 2 independent reviewers. Each app was assessed for general characteristics, professional context, functionality, quality, and privacy policy compliance using a previously published privacy policy compliance scale and the MARS.

**Results:**

A comprehensive analysis of the 115 identified apps revealed that all provided privacy policy links on their marketplace interfaces. Of these 115 apps, 104 (90.4%) displayed policy reminders during log-in, but only 85 (73.9%) implemented policy pop-ups or required active user confirmation. The average privacy policy compliance score across the 115 apps was 60.83% (SD 21.75%), with the highest average compliance in general characteristics (80.58%, SD 19.01%) and the lowest in information destruction (49.57%, SD 28.67%). Only 2 (1.7%) of the 115 apps notified third parties to promptly delete personal information. The mean MARS score was 3.41 (SD 0.26), indicating generally good app quality. A significantly positive correlation was found between MARS total scores and overall privacy policy compliance (*r*=0.354; *P*<.01), with the strongest associations observed for information destruction (*r*=0.405; *P*<.01) and data sharing or transfer (*r*=0.324; *P*<.01).

**Conclusions:**

Although privacy policy compliance in Chinese mental health care apps is at a moderate level, superficial personal information protections, inadequate implementation, and poor transparency in information destruction persist. The findings indicate that enhancing privacy policy compliance can significantly improve app quality and user engagement. Therefore, China’s internet content regulators should establish robust enforcement and oversight systems to strengthen the supervision of mental health care apps, elevating their privacy policy compliance to ensure the sustainable development of high-quality apps with enhanced privacy protection standards.

## Introduction

### Background

Globally, mental health problems such as depression and anxiety are becoming increasingly severe. Their prevalence has evolved into a public health challenge that cannot be ignored, with far-reaching effects on all aspects of social, professional, and personal life [1]. Nearly 1 billion people worldwide have a mental illness [2], and its disease burden ranks second among all global diseases [3], making it an important issue on the global health agenda. The latest report on the global economic burden of noncommunicable diseases predicts that by the beginning of 2030, mental illness will cause an additional US $16.1 trillion in losses and have a huge impact on global economic productivity and quality of life [4]. In the field of mental health services, the imbalance between the supply and demand of mental health services is particularly significant [5], with accessibility to high-quality mental health services severely lacking and a large number of patients not receiving effective treatment [6]. As a populous nation, China confronts significant challenges in the realm of mental health care. The lifetime prevalence of any mental disorder in China is 16.6% [7]. To illustrate, in Shanghai, while 21.4% of the population exhibits depressive symptoms, a mere 4.7% of these individuals seek mental health services [8]. The effective use of mental health services is a concern, which may be related to factors such as insufficient clinical public resources [9], a limited number of professionals [10], and an uneven geographic distribution of mental health services [11]. In addition, issues of safety, privacy, and the stigmatization of mental health services further hinder the effective use of mental health services [12,13]. Therefore, improving the accessibility, quality, and acceptance of mental health services has become a global issue that needs to be urgently addressed.

In this context, the emergence of mental health care apps as innovative intervention tools has significantly enhanced therapeutic accessibility through real-time availability, anonymity, personalized adaptation, and cost-effectiveness [14]. These apps typically incorporate cognitive behavioral therapy modules, mood tracking, mindfulness exercises, and artificial intelligence–driven chatbots. They demonstrated substantial potential during the COVID-19 pandemic [15], with evidence of high patient acceptance rates [16] and sustainable therapeutic outcomes [17,18]. However, the rapid proliferation of health apps has outpaced regulatory frameworks, resulting in the dissemination of substandard products [19,20] and complicating users’ ability to identify high-quality options. The absence of unified regulatory standards for health care app content remains a critical challenge [21]. In China, with its extensive mobile internet user base (1.092 billion internet users, with 99.9% accessing the internet via mobile phones as of December 2023) [22], mental health care apps present significant developmental opportunities. While governmental and health care systems have begun implementing oversight mechanisms to ensure data security and therapeutic efficacy, these digital solutions—despite their promise in improving mental health outcomes and strengthening psychiatric services [23-25]—also raise substantial privacy concerns that demand ongoing regulatory refinement to balance innovation with user protection.

At a time when the risk of personal information (PI) leakage is high, public concern about privacy protection is increasing, and a high-quality and well-developed privacy policy is an important consideration for users when choosing an app [26-28]. High-quality apps often pass strict testing and verification, and their functionalities have stability and security. A comprehensive privacy policy can enhance users’ trust in the app; reduce the legal risks caused by the violation of privacy protection regulations, such as PI misuse or leakage; and protect users’ legitimate rights and interests. Unlike other types of apps, mental health care apps store large amounts of highly sensitive PI, including mental health status, disease diagnosis, and emotional fluctuations, making private data leakage and security risks more concerning. Therefore, a clear, transparent, and data privacy– and security-compliant high-quality digital mental health care app is essential for enhancing user trust and promoting the effective use of mental health services.

The proliferation of mobile apps has catalyzed multidimensional advancements in privacy policy research. In terms of analytical tool development, the Polisis system proposed by Harkous et al [29] significantly improves automated privacy policy parsing efficiency, while the Mobile App Privacy System framework developed by Zimmeck et al [30,31] improves large-scale compliance evaluation capabilities. However, empirical research by Pan et al [32] on 10 mainstream automated privacy policy generators demonstrates that current technologies still exhibit significant deficiencies in risk identification accuracy and regulatory provision coverage. The literature review identifies 2 primary limitations in current research: first, most existing analytical frameworks are based on Western regulatory systems (eg, General Data Protection Regulation) and lack adaptation to China’s domestic legal framework; and, second, technical quality assessment and legal compliance requirements have not been systematically integrated. Therefore, this study innovatively combined the Mobile App Rating Scale (MARS) [33] with the privacy policy compliance criteria from the Information Security Technology–Personal Information Security Specification (PI Specification; GB/*t* 35273-2020), establishing for the first time a privacy policy evaluation system specifically for Chinese mental health care apps, aiming to fill research gaps in regional adaptability and domain specificity as well as provide evidence for enhancing industry privacy protection standards.

A series of privacy protection regulations issued in China (eg, the Code for Personal Information Security and the Law of the People’s Republic of China on the Protection of Personal Information) impose strict requirements on the collection, use, storage, and transfer of PI, which provide a standard basis for an app’s privacy policy. A systematic evaluation of privacy policy compliance in mental health care apps represents a critical frontline defense mechanism for protecting user data integrity and confidentiality. While existing research frequently addresses privacy evaluation as an ancillary aspect of app assessment, there persists a notable absence of methodologically rigorous frameworks with standardized evaluation protocols [34]. The MARS, despite being a validated instrument for quality appraisal [35-38], exhibits significant omissions in addressing core determinants such as data privacy safeguards, cybersecurity measures, and maintenance frequency—all indispensable components for holistic digital health product evaluation [39,40]. Shang et al [41] used the MARS to evaluate mental health care apps in China. However, recognizing the limitations of the MARS in assessing the privacy and security aspects of mobile health (mHealth) apps [42], Wu et al [43] conducted a supplementary descriptive statistical analysis focusing on whether apps claimed to protect privacy, as well as whether they displayed visible privacy indicators during use and reported compliance with relevant privacy regulations. However, this study did not examine the substance of privacy policies in depth and lacked an objective, specific, and systematic assessment of privacy policy compliance. Therefore, the impact of privacy policy compliance on overall app quality remains underexplored.

### Objectives

Building upon the aforementioned research context, this study uses a dual analytical framework integrating the MARS assessment system and the PI Specification compliance standards to systematically evaluate the quality and privacy policy compliance of mobile mental health care apps in China. The investigation seeks to address two pivotal research questions:

1. To what extent do the privacy policies of mental health care apps align with the PI Specification compliance standards?
2. What specific dimensions of privacy policy compliance correlate with app quality metrics?

## Methods

### Systematic Search Strategies

#### Overview

We assessed all mental health care apps available on the Android and iOS platforms. This study followed the PRISMA (Preferred Reporting Items for Systematic Reviews and Meta-Analyses) 2020 guidelines for systematic reviews [44]. The following subsections detail the search strategy, eligibility criteria, data extraction, quality assessment, and analysis methods.

#### Search Strategy

We conducted a systematic search for mental health care apps in Chinese app stores during March and April 2025. Unlike in most other countries, the Google Play Store remains inaccessible in mainland China [45]. Considering the popularity of Android smartphones in China [46], the 2 Android app stores with the largest market shares—Huawei AppGallery and Tencent Appstore—were selected to reflect the actual distribution channels in the country. Accordingly, we searched the Apple App Store (for iOS apps) and Huawei AppGallery and Tencent Appstore (for Android apps) [43]. Through a preliminary test search, the following keywords were identified: “psychology,” “mental health,” “mental healthcare,” “psychological counseling,” “psychological intervention,” “stress relief,” “emotions,” “depression,” and “anxiety.” Using their Chinese equivalents, we entered these keywords anonymously in the app stores, without logging in to any user account. All search results were collected to ensure that potentially relevant apps were comprehensively captured. If an app was available on both iOS and Android platforms with identical design and content, the Android version was selected. Empirical evidence [43,47] indicates that a minimum of 15 minutes per evaluator is required to adequately experience app functionalities while ensuring standardized and feasible assessments. Preliminary data show that the raters (XL and XW) took an average of 22.4 (SD 3.47) minutes per app (n=10) to complete quality and privacy policy compliance assessments. As 115 apps needed to be evaluated in this study, to balance efficiency with assessment quality, the time allocated for evaluating each app was set at 15 to 30 minutes.

#### Eligibility Criteria

In this study, we defined mental health care apps as digital tools that provide users with structured psychological interventions, symptom management, or therapeutic assistance through mobile devices or computer platforms. In alignment with the PI Specification*,* PI is statutorily defined as digitally or physically archived data elements capable of uniquely identifying individuals or characterizing behavioral patterns through singular or composite analysis. Such data encompass sensitive elements, including but not limited to names, government-issued ID numbers, biometric data, health-related physiological information, communication records, movement trajectories, and account credentials. We implemented sample screening and classification based on app titles and descriptions in the app stores. The inclusion and exclusion criteria are presented in Textbox 1.

**Inclusion and exclusion criteria.**

**Inclusion criteria**

- Targets the general population, excluding mental health professionals and corporate entities
- Provides content for individuals seeking professional psychological assistance, meeting at least one of the following core criteria:
  Professional interventions: includes psychotherapy modules supported by clinical guidelines or evidence-based practices (eg, cognitive behavioral therapy and dialectical behavior therapy techniques), symptom screening tools (eg, Patient Health Questionnaire-9 depression scale), or integration with professional medical services (eg, telepsychology consultations and electronic prescriptions)
  Disease specificity: specifically designed for intervening in and managing clinically diagnosed mental disorders (eg, depression, anxiety disorders, and posttraumatic stress disorder) or high-risk psychological states
  Dynamic adaptability: provides personalized feedback or real-time crisis intervention (eg, suicide risk alert systems) through user data analysis (eg, behavioral patterns and biometric indicators)
- Collects personal information at least once
- Available for free download
- Developed in or available in simplified Chinese

**Exclusion criteria**

- Functional irrelevance
  Apps lacking core mental health functionalities (eg, entertainment platforms, advertising tools, e-book readers, and social and dating platforms)
  Apps with content not grounded in scientific methodology, including pseudoscientific psychological elements (eg, astrology, tarot, and feng shui)
  Apps offering only generic tools (eg, mood diaries, meditation guides, and ambient audio) without targeted interventions
  Apps primarily designed for general health management (eg, sleep monitoring and menstrual cycle tracking)
- Nondirect user engagement
  Apps targeting mental health professionals or trainees (eg, clinicians, nurses, and counselors)
  Apps providing only indirect guidance (eg, content instructing parents on managing children’s psychological issues)
- Technical limitations
  Apps requiring supplementary electronic devices for core functionalities
  Apps with persistent technical malfunctions that impede normal operation
  Apps lacking accessible or complete privacy policy documentation
- Accessibility restrictions
  Apps requiring special credentials (eg, corporate or institutional accounts)
  Apps with mandatory payment for core functionalities, including those offering only time-limited free trials

After removing duplicates, each potentially eligible app was reviewed by 2 independent researchers based on app name, screenshots, and descriptions. All apps meeting the inclusion criteria were downloaded to test devices, with the reviewers archiving privacy policies as text files and recording download and update time stamps.

### Data Extraction and Content Assessment

Relevant information provided in the app marketplaces was extracted to assess the descriptive characteristics of each app. These general characteristics included platform, in-app purchases, developer, language, target audience, update time, privacy policy, functionality, and professional context. App characteristics and categories were documented by 2 independent researchers. All disagreements were resolved through discussion until consensus was reached.

### Privacy Policy Compliance Assessment

The privacy policy compliance scale developed by Ni et al [48] based on the PI Specification was used to assess the included apps. Finally, 6 level 1 indicators, 22 level 2 indicators, and 61 level 3 indicators were defined. Multimedia Appendix 1 presents a brief description of each indicator level, along with example sentences and the corresponding articles of the PI Specification. Each level 3 indicator was scored as 1 if the privacy policy complied with the criterion and as 0 otherwise. The score rate for each level 3 indicator was defined as the number of apps scoring 1 as a percentage of the total number of apps in the sample. The score for each level 2 indicator was calculated as the average of its associated level 3 indicators. The score for each level 1 indicator, which represents the compliance of an app at the corresponding stage of the PI life cycle, was calculated as the average of its associated level 2 indicators. For each app, the sum of the scores across all indicators was converted to a percentage, representing the app’s overall privacy policy compliance score.

Before conducting the privacy policy compliance assessments, the 2 raters (XL and XW) received systematic training from professional IT developers to ensure that they possessed the necessary technical and legal knowledge for evaluating privacy policy compliance. The training covered key aspects of privacy policies (eg, data collection, storage, and sharing), the interpretation of common IT and legal terminology, and standardized scoring procedures. In a prestudy calibration phase, both evaluators independently assessed 10 mobile apps, achieving an exceptional intraclass correlation coefficient (ICC) of 0.996 (95% CI 0.983-0.999) for interrater reliability. Discrepancies were resolved through systematic consultations between the raters and technical experts to establish unified criteria. After this standardization process, the raters independently evaluated all included apps. A subsequent reliability analysis yielded an ICC of 0.988 (95% CI 0.982-0.991) for the privacy policy compliance assessments.

### Quality Assessment

The quality of the included apps was assessed using the MARS [33]. As a widely used tool for evaluating the quality of health-related mobile apps, the MARS has been applied to assess apps focused on pharmaceuticals [45,49], disease management [50-53], health behavior management [47,54,55], and mental health [56-60]. It consists of 23 items with 4 objective quality subscales (engagement, functionality, aesthetics, and information quality) and 1 subjective quality subscale. Each item is assessed on a 5-point Likert scale ranging from 1 (inadequate) to 5 (excellent). Given our focus on objective app quality, we excluded the subjective quality subscale from our analysis. The rating procedure strictly adhered to the standardized implementation guidelines established in the original validation study [61]. Two raters completed systematic training on MARS administration to ensure consensus understanding of the instrument’s structure and assessment dimensions. During the pilot phase, 2 independent reviewers screened out apps deemed to have insufficient validity, with the MARS demonstrating good internal consistency (Cronbach α=0.903, 95% CI 0.555-0.973). For any unresolved disagreements, a third researcher was invited to arbitrate. This arbitrator possesses experience in developing digital health management tools, holds 5 software copyrights, and has published 3 academic papers in the field of mHealth apps (including 1 paper indexed in the Science Citation Index Expanded and 2 Chinese core journal papers), thereby ensuring a unified understanding of MARS items and evaluation criteria.

To ensure methodological rigor, ICCs based on a 2-way random effects model (using absolute agreement and mean measurement calculations) were used to rigorously evaluate interrater reliability for domain and total MARS scores, thereby enhancing the validity and scientific credibility of the assessment outcomes [62].

### Data Analysis

Data were analyzed using SPSS software (version 27.0; IBM Corp). Categorical variables from the content assessment were described using frequencies and percentages. Quantitative variables from the quality and privacy policy compliance assessments were described using means and SDs to describe trends in the concentration and dispersion of the data. Pearson correlation analyses were used to compare MARS and privacy policy compliance scores. *P* values <.05 were considered to be statistically significant.

### Ethical Considerations

This study obtained approval from the Ethics Committee of Fujian Provincial Hospital (K2021-03-029). However, the current manuscript represents a systematic evaluation of mHealth applications and does not involve direct human subject participation, intervention, or primary data collection.

As this research exclusively analyzed publicly available app features and privacy policies, no additional ethics review or informed consent was required per institutional guidelines. All app information was obtained from open sources (eg, official app stores and developer websites), and no user data were accessed or processed.

## Results

### Overview

**Figure 1:**
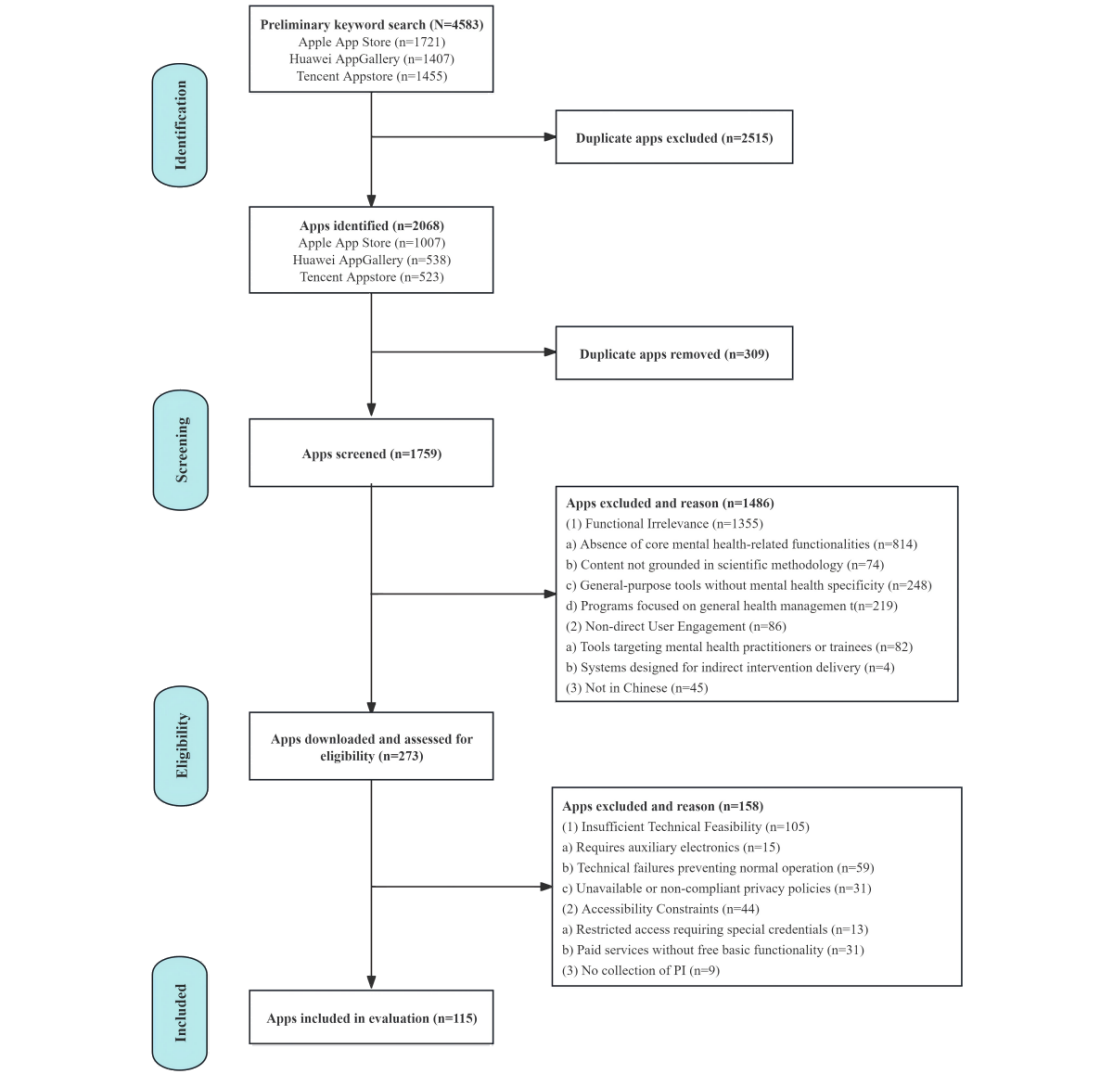
Flowchart illustrating the systematic search and selection process of mental health care apps. PI: personal information.

We systematically screened mental health care apps across major Chinese mobile platforms (Apple App Store, Huawei AppGallery, and Tencent Appstore) using predefined keywords for both iOS and Android systems. The initial search yielded 4583 potentially relevant apps, which were reduced to 2068 (45.12%) unique apps after deduplication within individual platforms (Apple App store: n=1007, 48.69%; Huawei AppGallery: n=538, 26.02%; and Tencent Appstore: n=523, 25.29%). Of these 2068 apps, cross-platform comparison subsequently excluded 309 (14.94%) duplicate apps. Of the remaining 1759 apps, 1486 (84.48%) were excluded based on the inclusion and exclusion criteria, and 273 (15.52%) were downloaded to the evaluation device for further assessment. During the download evaluation phase, of the 273 apps, 60 (22%) were excluded due to technical incompatibility (installation failure or operational abnormalities), 9 (3.3%) for lacking personal data collection features, 31 (11.4%) for inaccessible privacy policies, and 13 (4.8%) for requiring institutional verification codes (enterprise, school, or medical access only). Ultimately, 115 (42.1%) of the 273 apps met all study criteria and were included in the final analysis (Figure 1).

### Content Assessment of Included Apps

Of the 115 apps, 27 (23.5%) were sourced from the Apple App Store, 62 (53.9%) from Huawei AppGallery, and 26 (22.6%) from Tencent Appstore. The majority of the apps (110/115, 95.7%) were developed by companies, of which 13.6% (15/110) specialized in the development of mental health care apps. Of the remaining 5 apps, 4 (3.5%) were sourced from mental health service institutions, and 1 app (0.9%) was developed by an individual developer. Functional analysis revealed that apps with multimodule integration constituted the predominant category (106/115, 92.2%). Only a few apps were completely free (20/115, 20.9%), and most required an additional service fee (91/115, 79.1%). In addition, of the 115 apps, 1 (0.9%) was specifically designed for children or adolescents, and 2 (1.7%) served as diagnostic and treatment platforms for patients with mental health issues. In terms of update frequency, of the 115 apps, 35 (30.4%) apps had been updated within the past month, 55 (47.8%) had been updated within the past year, and 20 (17.4%) had not been updated for more than a year. Table 1 summarizes the basic characteristics of the included apps.

**Table 1:** Descriptive data of the included apps (n=115).

Characteristics		Apps, n (%)
**Platforms**		
	Apple App Store	27 (23.5)
	Huawei AppGallery	62 (53.9)
	Tencent Appstore	26 (22.6)
**In-app purchases**		
	Yes	91 (79.1)
	No	24 (20.9)
**Developers**		
	Companies	110 (95.7)
	Psychological services organizations	4 (3.5)
	Individuals	1 (0.9)
**Functionality**		
	Single function	9 (7.8)
	Multifunctional	106 (92.2)
**Target audience**		
	Children or adolescents	1 (0.9)
	General public	112 (97.4)
	Patients with mental health issues	2 (1.7)
**Last update time**		
	<1 mo	35 (30.4)
	≥1 mo to ≤1 y	55 (47.8)
	>1 y	20 (17.4)

Regarding privacy policies, it is notable that all apps provided a link to access their privacy policies in their marketplace interfaces. Of the 115 apps, 104 (90.4%) displayed a privacy policy notification at log-in; however, only 85 (73.9%) featured a separate pop-up or required users to click to confirm consent. Particularly concerning, only 22 (19.1%) of the 115 apps included a privacy policy section on the personal home page, while the vast majority (85/115, 73.9%) provided a privacy policy link in their system settings.

In the app marketplace descriptions, 23 (20%) of the 115 apps did not mention any relevant professional background regarding the design of their functionality. Of the 115 apps, 7 (6.1%) incorporated established psychological treatment theories such as cognitive behavioral therapy; furthermore, 60 (52.2%) apps enabled access to input from professionals certified in mental health, and certain features (such as depression and anxiety tests) in 72 (62.6%) apps were supported by academic research.

**Figure 2:**
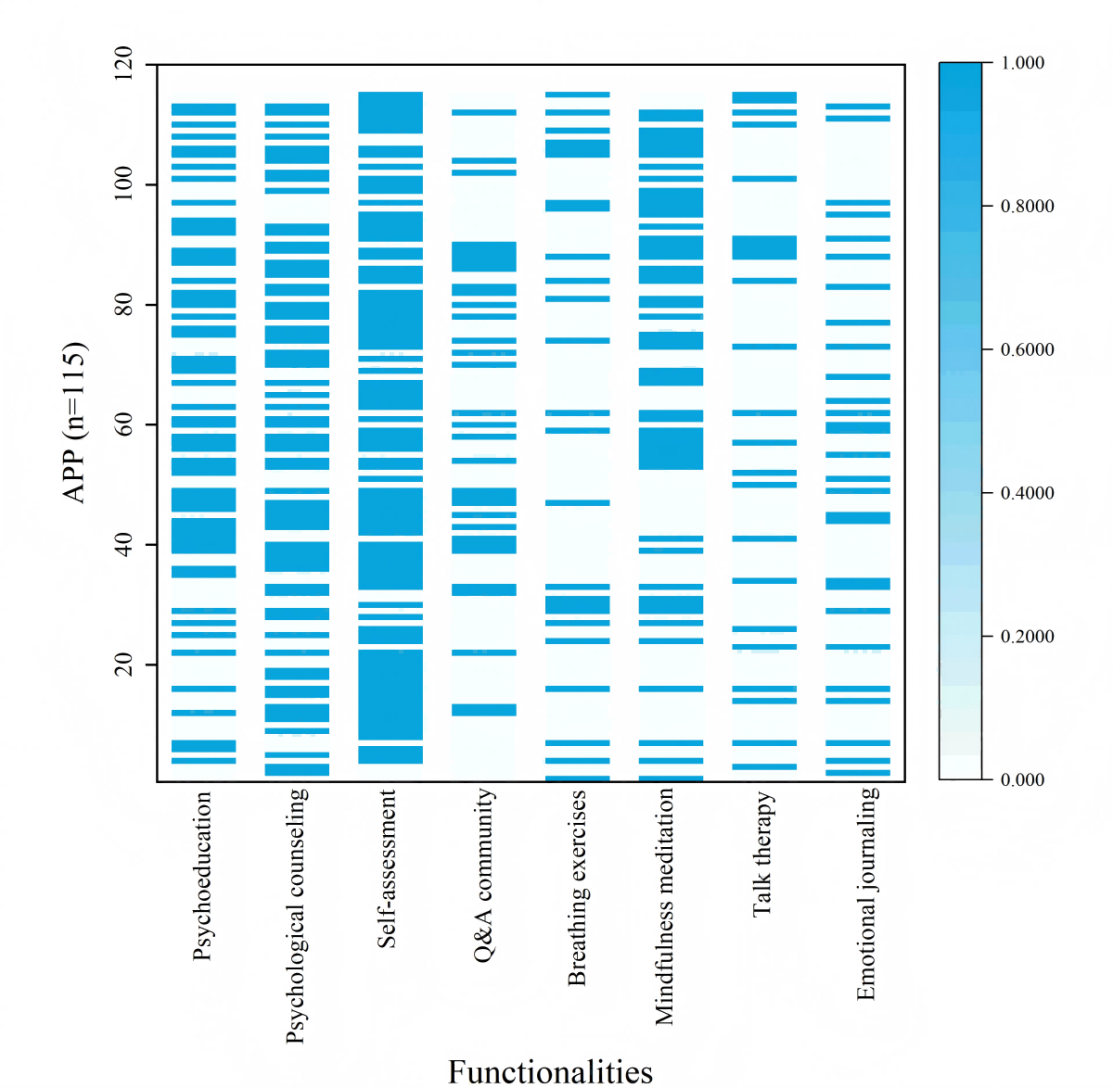
Heat map depicting the distribution of functionalities across the apps. The color gradient indicates the presence (blue, encoded as 1) or absence (white, encoded as 0) of specific functionalities. Each row represents an app’s functional profile, demonstrating the binary distribution of functionality presence. Q&A: question-and-answer.

Psychological counseling (66/115, 57.4%) and psychological assessments (88/115, 76.5%) were the most commonly featured functions, while breathing exercises (25/115, 21.7%), talk therapy (23/115, 20%), and emotional journaling (28/115, 24.4%) were less frequently observed (Multimedia Appendix 2). The most common combination of functionalities was psychological counseling, psychological assessments, and psychoeducation (34/115, 30.4%), followed by psychological counseling, psychological assessments, and mindfulness meditation (23/115, 21.7%), as illustrated in Figure 2.

### Privacy Policy Compliance Assessment of Included Apps

**Figure 3:**
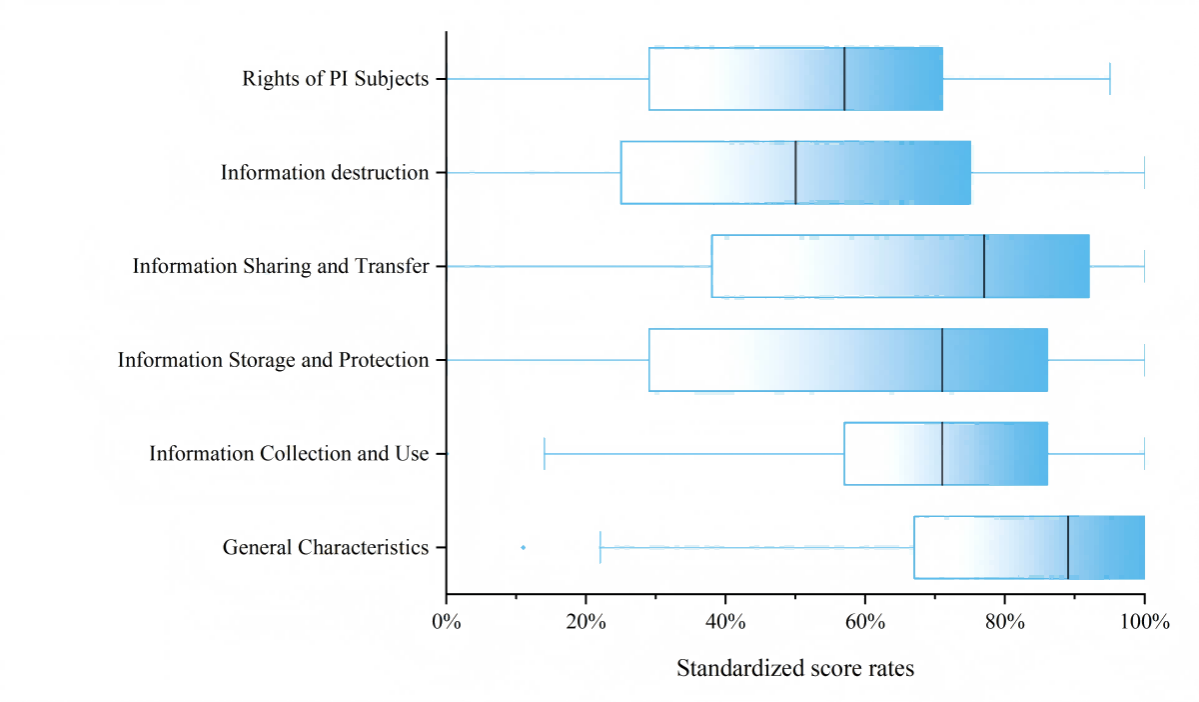
Score rates for mental health care apps on level 1 indicators. The boxes in the box plots depict IQRs, with whiskers extending to 1.5×IQR for outlier detection. score rate. Median values are marked by vertical lines within each box. PI: personal information.

The average privacy policy compliance score for the 115 apps was 60.83% (SD 21.75%), indicating a very high degree of dispersion. The results of the assessment of the level 1 privacy policy indicators are shown in Figure 3. The most compliant items were general characteristics (80.58%, SD 19.01%), information collection and use (67.33%, SD 22.77%), and information sharing and transfer (67.09%, SD 27.73%). However, overall compliance was lower for some items, such as information destruction (49.57%, SD 28.67%), information storage and protection (50.14%, SD 27.6%), and the rights of individuals whose PI was collected (55.78%, SD 29.07%). The name of each app along with the results of the assessment are provided in Multimedia Appendix 3.

The average score rate for level 2 indicators was 58.16% (SD 17.40%; range 16.09%-86.09%). We used bar charts to visualize the results of the evaluation of the degree of privacy policy compliance. In each bar chart, the vertical axis presents the scoring indicators (including level 3 and level 2 indicators) and the score rates of level 2 indicators; the horizontal axis indicates the score rates of level 3 indicators. To more intuitively reflect the scores of level 2 indicators, we used different colors to visually represent each level 2 indicator: yellow for values near the overall average, red for values near the minimum, and green for values near the maximum.

The general characteristics of a privacy policy reflect its openness, readability, and the timeliness of updates. The results of the compliance evaluation of the general characteristics of privacy policies are shown in Multimedia Appendix 4. The 3 subindicators under core characteristics demonstrated relatively higher compliance rates: policy updates (71.88%), app scope (83.77%), and policy disclosure (86.09%). Although all apps maintained separate and easy-to-access privacy policies, only 66.1% (76/115) provided systematic documentation through organized frameworks with navigable content summaries.

The results of the compliance assessment for the information collection and use phase and the information storage and protection phase are shown in Multimedia Appendices 5 and 6. During the information collection and use phase, the score for sensitive PI (56.09%) fell below the average for this section, indicating a relatively low level of compliance. While 80% (92/115) of the apps asserted that they obtained explicit consent when collecting information from minors, only 32.2% (37/115) prominently labeled sensitive PI in a conspicuous manner.

While the compliance rate for information storage security (61.52%) surpassed the benchmark (55.78%), only 18.3% (21/115) of the apps disclosed their implemented security protocols and certifications to individuals whose PI was collected. Moreover, although 64.3% (74/115) of the apps described notification procedures after security incidents for individuals whose PI was collected, and 59.1% (68/115) pledged accurate reporting to regulatory bodies, less than one-third (24/115, 20.9%) explicitly accepted legal responsibility for privacy-related security incidents.

Multimedia Appendix 7 shows the results of the compliance assessment for the information-sharing and transfer phase and the information destruction phase. In terms of PI sharing, the compliance score for the indicator assessing apps’ communication of security measures before sharing was relatively low, with only 64 (55.7%) of the 115 apps meeting this criterion. The majority of the apps (64/115, 55.7%) reported implementing measures such as anonymization or deidentification of PI through IT processes, rendering the information untraceable to the individual concerned. The score for data deletion and anonymization during the information destruction phase was below average (49.57%). Worryingly, only 1.7% (2/115) of the apps indicated that they notified third parties regarding the timely deletion of the corresponding PI.

There were significant differences between the scores of indicators related to the rights of individuals whose PI was collected (Multimedia Appendix 8); particularly noteworthy was the score for the secondary indicator concerning the right to obtain a copy of PI (16.09%), which was significantly below the average. Specifically, only 16.5% (19/115) of the apps addressed the right to obtain a copy of PI, and just 15.7% (18/115) provided a method for doing so, which suggests that the majority of apps fail to prioritize guaranteeing users the right to obtain a copy of PI.

### Quality Assessment of Included Apps

The interrater reliability of the included apps’ quality assessment was high, with the MARS scores overall showing high correlation (ICC=0.968, 95% CI 0.953-0.978). In addition, all subscales showed good agreement: engagement, ICC=0.949 (95% CI 0.927-0.965); functionality, ICC=0.956 (95% CI 0.937-0.970); aesthetics, ICC=0.918 (95% CI 0.880-0.943); and information, ICC=0.957 (95% CI 0.954-0.978). The internal consistency of the MARS total and domain scores was considered excellent.

The mean overall MARS score was 3.41 (SD 0.26; range 2.75-4.22), indicating a moderate overall quality. Of the 115 apps, 3 (2.6%) scored ≥4.00 on the MARS. Furthermore, of the 115 apps, 105 (91.3%) had scores of between 3.00 and 3.93, while 7 (6.1%) attained scores ranging from 2.75 to 2.98. No app received a score of <2.00.

**Figure 4:**
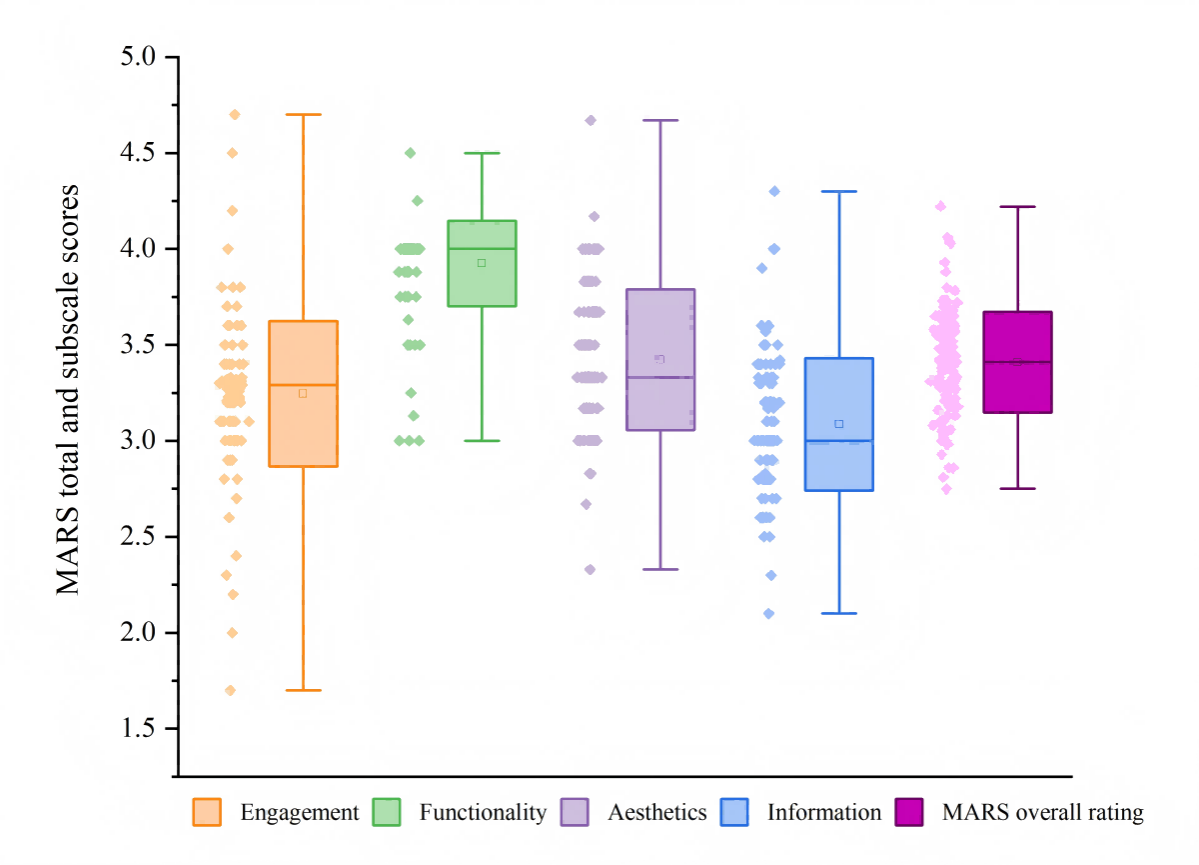
Distribution of the Mobile App Rating Scale (MARS) total and subscale scores (n=115). Values are presented as median (range), mean, and SD. The whiskers represent the data range.

The highest scoring domain was functionality, followed by aesthetics, engagement, and information. The average scores for each subscale were as follows: functionality quality, 3.92 (SD 0.22); aesthetics quality, 3.42 (SD 0.37); engagement quality, 3.25 (SD 0.38); and information quality, 3.08 (SD 0.35). The range of scores for engagement quality was the broadest (1.70-4.70), while the functionality quality scores had the narrowest range (3.00-4.50). Figure 4 illustrates the distribution of overall quality scores and scores across the 4 subscale dimensions.

### Correlation Analysis of Privacy Policy Compliance With the MARS

**Figure 5:**
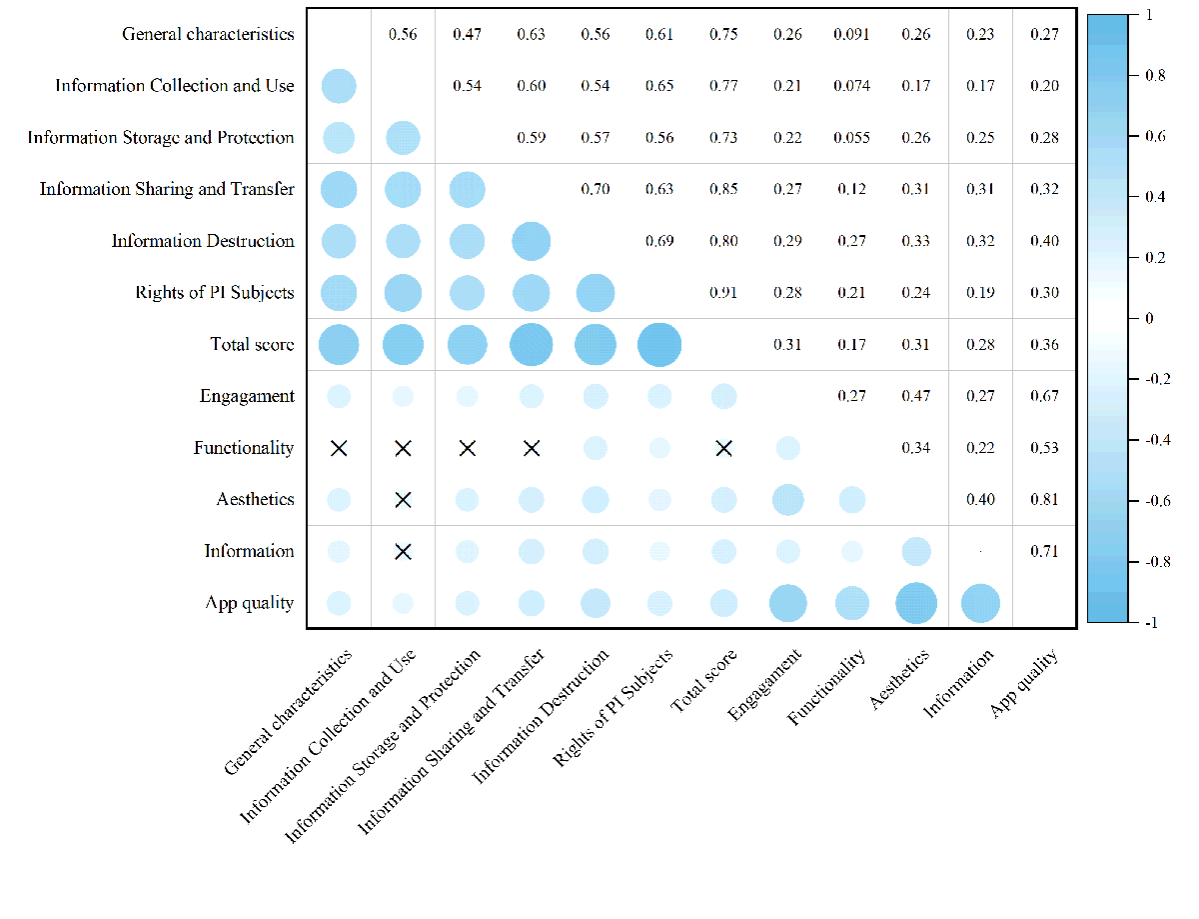
Correlation analysis of mental health care app Mobile App Rating Scale scores and privacy policy compliance (n=115). The crosses indicate that the correlation is not statistically significant. PI: personal information.

The correlation analysis results between MARS scores and privacy policy compliance are shown in Figure 5. The total MARS score demonstrated a significant positive correlation with overall privacy policy compliance (*r*=0.354; *P*<.01). Information destruction (*r*=0.405; *P*<.01) and information sharing and transfer (*r*=0.324; *P*<.01) showed the strongest associations with the total MARS score. Among the MARS dimensions, user engagement showed significant positive correlations with all privacy policy categories (*r*=0.204-0.294; *P*<.05). Aesthetics and information quality were only insignificantly associated with the information collection and use domain, while functionality emerged as the sole dimension without significant association with overall compliance (*r*=0.174; *P*>.05).

## Discussion

### Principal Findings

This study identified 115 mental health care apps to examine their general characteristics and to assess their content, quality, and privacy policy compliance.

The sample demonstrated a marked underrepresentation of programs serving children and adolescents (1/115, 0.9%) and populations with psychiatric illnesses (3/115, 2.6%). Helping Minds–CBT to Overcome Anxiety and Depression, Good Mood, and Doctor Zhaoyang Patient Edition were the only 3 apps specializing in specific mental disorders. The first focused on depression and anxiety management, while the latter two addressed broader psychiatric conditions, such as schizophrenia and bipolar disorder, aligning with the findings reported by Wu et al [43]. Notably, regarding PI collection, only Good Mood required users to submit identity documents and emergency contact information. This configuration likely stems from mental health services’ unique risk management mechanisms: identity verification ensures the biometric accuracy of user information, while emergency contacts facilitate rapid connection with designated responders during crises or risk-indicating self-assessments, which can reduce the time window between crisis identification and intervention.

Regarding development backgrounds, only 4 (3.5%) of the 115 apps originated from mental health institutions, while 110 (95.7%) were developed by companies, of which 15 (13.6%) specialized in mental health care apps. This suggests that most mental health care apps may lack credible development backgrounds, potentially having been created by individuals without mental health training. Giunti et al [63] noted that most existing apps were developed by non–health care professionals who, despite design creativity and technical skills, lacked essential scientific knowledge. The absence of psychological expertise in app development may compromise credibility [43]. Furthermore, nonpsychological professionals may inadequately select and apply psychological methodologies, resulting in content and functional deficiencies and questionable app validity, posing potential user risks [64]. These issues underscore the necessity for psychological expertise in development teams to ensure the scientific validity, effectiveness, and sustainability of mental health care apps.

In health care, privacy is of paramount importance, and the lack of privacy policies has raised concerns about the legitimacy of the “I agree” option presented to users [65]. Surveys have indicated that apprehension about personal data collection is a frequently cited reason for rejecting health care app adoption [66,67]. Legal frameworks have historically recognized the privacy and confidentiality of personally identifiable information as fundamental human rights [68]. Among 79 certified safe and reliable health apps in the UK National Health Service Apps Library, 66% lacked encryption during PI transfer, while 20% completely lacked privacy policies [69]. By contrast, preliminary screening in this study revealed that 88.6% (242/273) of the apps included privacy policies. This finding aligns with that of Wu et al [43], who assessed multifunctional mental health care apps in China, demonstrating the enhanced prioritization of privacy policies in domestic mental health care apps.

An in-depth analysis of privacy policy compliance found that 20.9% (24/115) of the apps scored <40%, with a mean score of 60.83% (SD 21.75%), indicating that most Chinese mental health care apps are in a preliminary compliance stage regarding PI regulations. This mean score exceeds the mean compliance scores of 40.4% reported by Ni et al [48] for chronic disease management apps and 59.9% identified by Jiang and Zheng [70] for provincial health code apps. The observed divergence could be attributed to heterogeneity in app categories, assessment methodologies, regulatory landscapes, and progressive policy prioritization at the national level. Notably, significant challenges persist regarding the security and transparency of PI collection [71,72], although apps targeting specific audiences demonstrate better privacy and security performance [73]. Professional mental health institutions or specialized app developers generally maintain robust privacy compliance systems, whereas general commercial or technology companies often lack effective control mechanisms, raising doubts about policy efficacy and security [74], potentially explaining the wide score dispersion observed in this study.

Notably, the content of privacy policies in current mental health care apps exhibits considerable homogenization with that of privacy policies in general apps, with approximately two-thirds of the policies being generic [75]. This phenomenon may stem from developers’ widespread adoption of standardized privacy policy templates that focus solely on common type of personal data (eg, names and email addresses), while failing to adequately address the heightened sensitivity of mental health–related PI (eg, anxiety, depression, or other mental health conditions) [15]. Therefore, future developers should formulate differentiated privacy policies based on the apps’ specific functionalities and legal requirements, providing explicit specifications for the collection, use, protection, and destruction of sensitive health information. At the same time, regulatory authorities should establish dynamic security evaluation systems targeting mental health care apps because these systems are crucial for identifying privacy risks in data collection and storage processes [15], thereby enhancing industry-wide data protection standards, optimizing user experience, and strengthening user trust.

In terms of general characteristics, policy disclosures and updates are fundamental prerequisites for effectively safeguarding the legitimate rights and interests of individuals whose PI is being collected. Most level 3 indicators in these categories scored between 66.09% and 100%, indicating that most PI controllers possess a basic awareness of user privacy protection. Nevertheless, only 58.3% (67/115) of the apps explicitly stated that privacy policy updates did not necessitate reobtaining consent from those whose data were collected, which may contribute to mHealth app abandonment stemming from health information security apprehensions [76,77]. Furthermore, only 66.1% (76/115) of the apps demonstrated coherent organizational frameworks with content indices, consistent with the observations by Ni et al [48]. Crucially, despite widespread emphasis on timely updates in privacy policies, notification methods remain ambiguous; for instance, A Journey of the Mind stated “we will notify you of new Privacy Policy during version updates,” while A Psychological Helper declared that “updated terms immediately supersede previous versions without separate notice,” highlighting transparency deficiencies in policy communication.

In data collection and use, 80% (92/115) of the apps required explicit consent for acquiring information from minors, significantly exceeding the rates reported in previous studies [43,70], indicating industry progress in protecting minors’ PI. However, only 15.7% (18/115) of the apps independently provided documents such as “Children’s Personal Information Protection Guidelines” or “Children’s Privacy Policy Statement.” Among these, Glowe Attic notably emphasized “Guardian Notice” requirements. Concerning sensitive data acquisition, only 32.2% (37/115) of the apps fully implemented regulatory requirements in privacy governance, leaving the majority potentially heightening exposure risks for users experiencing psychological vulnerability (eg, individuals with severe anxiety or depressive disorders) [65]. In the case of inappropriate data sharing, leakage, or destruction, users may face serious negative consequences [78].

The sensitive nature of psychological health data mandates rigorous safeguards throughout information-sharing procedures. Compared with previous studies, the industry has made substantial progress in data transparency: >80% (103/115) of the apps in this study described data-sharing end points (including types of shared information, purposes, and data recipient categories) [79]. However, only 55.7% (64/115) detailed security measures implemented before PI sharing. More concerningly, most of the apps demonstrated poor compliance in entrusted processing (33/115, 28.7%) and cross-border transfers. Related research indicates that only one-third of participating enterprises claimed that they would not sell user PI without consent, while a minority admitted selling non-PI or aggregated information [78]. Concurrently, information-sharing policies frequently suffer from ambiguous explanations or excessive complexity, particularly regarding protected health information and third-party sharing, creating user comprehension barriers [17]. Therefore, this remains a significant challenge as far as the practice of information sharing is concerned.

Information storage and information protection constitute critical components of privacy safeguards. In this study, more apps explained their PI storage practices compared to prior research (48.48%) [80]; however, only 67.8% (78/115) described organizational-level security measures. A mere 18.3% (21/115) explicitly disclosed PI security protocol certifications, with 8 (38%) of these 21 apps specifically stating that they had “completed National Information Security Level Protection (Grade 3) registration and evaluation, and obtained certifications including ISO 27001 Information Security Management System.” This indicates that most apps fail to meet national certification standards, exhibiting significant deficiencies in data protection measures that may increase the risk of information leakage. Related research shows that while one-fourth of apps mentioned implementing security measures to protect user data, most apps explicitly disclaimed guarantees of data security [80]. A striking 79.1% (91/115) of the included apps failed to clarify legal responsibilities in PI security incidents, reflecting prevalent industry practices of legal circumvention: numerous health care apps self-identify as “wellness tools” to exploit regulatory loopholes and evade or mitigate legal liabilities in user data breaches [79]. Such practices not only undermine the authority of legal frameworks but also underscore the urgent need to strengthen regulatory oversight and enhance corporate self-governance [81]. China’s current legal regulatory framework for apps remains incomplete. It is primarily governed by the Cybersecurity Law, which focuses on protecting basic PI and standardizing data storage methods [82] but lacks unified regulatory mechanisms and specific enforcement standards. Moving forward, collaborative efforts among government regulators, developers, health care professionals, and the public are essential to establish a more robust and transparent regulatory system [83]. Such a system would ensure that all apps comply with standardized requirements to effectively safeguard users’ information security and legal rights.

Information destruction constitutes a weak link in privacy policy implementation. The poorest-performing aspect was the requirement to notify third parties for timely PI deletion, with only 2 (1.7%) of the 115 apps (Slow Language Space and Doctor Zhaoyang Patient Edition) including statements such as “while responding to individual deletion requests, [we] endeavor to notify third parties that obtained PI from controllers and require prompt deletion.” This figure is significantly lower than the 21% reported in the study by Ni et al [48]. The results of a study conducted in Germany indicated that only slightly more than half of email requests to app providers to delete information were fulfilled [80]. By contrast, our analysis demonstrated that 78.3% (90/115) of the included apps implemented proper PI destruction or anonymization after account termination. Regulatory mandates (articles 6.1 and 8.5 of the PI Specification) require data controllers to implement PI deletion and anonymization upon retention period expiration or user account deactivation. Nevertheless, excessive data retention durations and service disruptions stemming from inadequate storage protocols remain prevalent issues [79]. Although 64.3% (74/115) of the included apps claimed legal compliance in PI retention periods, only 31% (23/74) specified concrete storage durations, and merely 16% (12/74) cited foundational laws such as the E-Commerce Law or the Cybersecurity Law. These findings reveal deficiencies in standardized operational procedures and delayed information updates.

Regarding the protection of the rights of individuals whose PI is collected, studies suggest that recognizing PI as a right positively contributes to its protection [84]. However, our findings reveal that while most of the included apps declared that individuals whose PI was collected had the right to access their PI and withdraw consent, they inadequately specified the operational methods for exercising these rights; for instance, 74.8% (86/115) of the apps declared a right to access PI; yet, only 60.9% (70/115) specified access methods, consistent with the study by Wu et al [43], who found that only 42.4% of apps enumerated user data management rights. Furthermore, some privacy policies section headers mention “right to PI deletion” but leave the corresponding content sections blank. These findings indicate a discrepancy between privacy policy content and actual functionality in most Chinese mental health care apps, which aligns with the research by Song et al [85]. The protection of the rights of individuals whose PI is collected in Chinese apps tends to be superficial, failing to substantively implement user rights. Regarding account cancellation, 80.9% (93/115) of the included apps acknowledged users’ right to delete accounts; yet, only Glowe Attic implemented a 7-day cooling-off period after a deletion request.

Privacy policy compliance significantly impacts app quality. Our findings reveal a significant positive correlation between privacy policy compliance and app quality (*r*=0.354; *P*<.01), with information destruction as well as information sharing and transmission showing the strongest correlations, providing robust evidence for the critical role of privacy policies in enhancing app quality. At present, users are highly concerned about privacy risks, such as those related to data processing and cross-platform or cross-border information sharing and transmission, and demand greater transparency and stronger privacy protections [86]. Notably, studies indicate that higher download volumes correlate with elevated privacy scores, suggesting a positive feedback loop between user preference and privacy compliance [87]. Apps that follow the principles of privacy protection and strictly comply with relevant laws and regulations are more likely to win the trust of users and the favor of the market [88]. Conversely, policy ambiguity or inaccessibility may erode user security perceptions, negatively impacting overall app evaluations and use intentions [89]. Therefore, PI controllers must prioritize mechanisms for PI sharing, transfer, and deletion by clearly defining PI purposes, exhaustively listing recipients (including third-party providers), and specifying sharing rationales (including personalization, feature optimization, and market research) and information destruction measures (such as deleting or anonymizing PI after account cancellation and notifying third-party platforms to delete PI) to strengthen trust as well as foster engagement and loyalty, thereby elevating overall app quality [90].

Existing studies show that 1 reason users are reluctant to download certain apps is their lack of comprehensive privacy policies, while inadequate privacy protections may also lead to app discontinuation after installation. Therefore, improving privacy policy compliance may help enhance user engagement. Specifically, privacy policies covering PI collection and use rules, information storage and security measures, data-sharing and transfer methods, and explanations of the rights of individuals whose PI is collected can significantly reduce users’ concerns about privacy leaks, thereby alleviating the current widespread problems of high app abandonment and low continued use rates [17]. However, there are dual challenges in reality: software developers’ insufficient legal knowledge regarding app development and the poor readability of policy texts. On one hand, not all developers possess adequate legal expertise, resulting in omissions or ambiguous expressions in privacy policies. On the other hand, existing studies confirm that understanding privacy policies requires relatively high reading ability, and overly complex or lengthy texts can create comprehension barriers for users, increase their information anxiety, and ultimately reduce their willingness to participate [15,88,91]. These dual obstacles of insufficient legal professionalism and poor text readability jointly undermine the trust-building function that privacy policies should perform. Thus, software developers should consult legal experts for policy formulation, adopt standardized privacy policy frameworks, and add visual explanations to enhance policy transparency and user understanding, thereby optimizing user participation experience.

Notably, no significant correlation was found between MARS functionality and overall privacy policy compliance scores (*r*=0.174; *P*>.05), which may be related to software developers’ focus on user behavior metrics in mHealth apps [92]. Consequently, developers tend to prioritize improving functional metrics such as app performance and user interface logic, while paying less attention to privacy policy compliance. Similarly, aesthetic dimensions (such as interface layout, graphic quality, and visual appeal) and information quality dimensions (such as content accuracy and source reliability) in the MARS assessment lacked significant correlation with the collection and use of PI in privacy policies, which reflects the relative independence between an app’s functional design and its privacy policies. Several interpretative frameworks help elucidate this observation: first, visual design and information quality primarily affect users’ direct use experience and interface interaction perception, while privacy policies serve as institutional safeguards for user trust and data protection compliance, representing fundamentally different core concerns. Research shows that most apps’ consent process consists merely of an initial screen displaying the privacy policy and an “I agree” button, with developers minimizing steps in the onboarding process to reduce friction and improve user experience, while neglecting privacy policy safeguards [15]. Balancing privacy and user experience remains challenging and requires further investigation [15]. Second, the complexity and readability issues of privacy policy texts exacerbate users’ difficulties in understanding privacy content, thereby affecting the actual effectiveness of privacy policies [93]. Improvements in aesthetics and information quality alone may not resolve this core issue. Therefore, future development and evaluation of mental health care apps should emphasize the synergistic integration of privacy protection and user experience to ensure potential users’ trust and enhance overall app quality.

### Limitations and Future Work

First, although we assessed the privacy policy compliance of mental health care apps, our evaluation metrics were exclusively grounded in the PI Specification, without incorporating standards from the 2021 Personal Information Protection Law. Consequently, we could not demonstrate the evolution of privacy policy compliance under the combined regulatory framework and mandatory provisions of both.

Second, our investigation focused on evaluating apps’ adherence to regional legal statutes, as opposed to directly measuring the effectiveness of user privacy safeguards.

Third, the evaluation time for each app’s quality and privacy policy was limited to 15 to 30 minutes, which might have led to oversight of certain specific content due to time constraints.

Fourth and last, due to funding limitations, we did not include paid apps or those offering only limited free trials, potentially overlooking differences between free and paid apps regarding MARS ratings and privacy policy compliance.

In addition, some of our findings suggest directions for future research.

Given the scarcity of privacy policy compliance assessment tools and the need to closely follow dynamic changes in national laws and regulations, there is an urgent need to develop or update privacy policy evaluation scales with legal authority, high validity, and standardization. The development of such scales should not only reflect legal consensus but also ensure their broad applicability and accuracy in practice to cope with increasingly stringent legal regulatory environments.

Moreover, because current privacy policies are primarily based on national laws and regulations—resulting in variations in content, details, and emphasis—and due to the absence of specific evaluation standards for mHealth app privacy policy compliance, there is an urgent need to establish a universal privacy policy assessment framework to meet evaluation needs across different countries.

### Conclusions

This study systematically identified and analyzed 115 mental health care apps; assessed their content, quality, and privacy policy compliance; and provided a scientific evaluation framework as well as insights for digital tools in the mental health field. Overall, China’s mental health care apps demonstrate good quality and offer easily accessible privacy policies, but significant gaps remain in implementing key indicators, highlighting the urgent need for PI protection and the necessity for enhanced national regulation and compliance review. This study highlights that to enhance user trust and engagement, developers should create and implement privacy policies that are clear, transparent, and consistent with users’ expectations, especially with regard to information sharing and transfer. This provides theoretical support for the future development of mental health care apps, aiming to promote both industry standardization and increased user engagement.

## Appendix

Multimedia Appendix 1 Evaluation indicators and guide for privacy policies.

Multimedia Appendix 2 Frequency distribution by app functionality type.

Multimedia Appendix 3 List of mental health care app names and evaluation results.

Multimedia Appendix 4 Compliance evaluation results for general characteristics of privacy policies.

Multimedia Appendix 5 Compliance evaluation results for the information collection and use stage.

Multimedia Appendix 6 Compliance evaluation results for the information storage and protection stage.

Multimedia Appendix 7 Compliance evaluation results for the information sharing and transmission as well as information destruction stages.

Multimedia Appendix 8 Compliance evaluation results for the rights of individuals whose personal information was collected.

## Abbreviations

ICC: intraclass correlation coefficient
MARS: Mobile App Rating Scale
mHealth: mobile health
PI Specification: Information Security Technology–Personal Information Security Specification
PI: personal information
PRISMA: Preferred Reporting Items for Systematic Reviews and Meta-Analyses
