# Supplementary figures and images for: Quality and Privacy Policy Compliance of Mental Health Care Apps in China: Cross-Sectional Evaluation Study
Source: J Med Internet Res. 2025 Jul 3;27:e66762. doi: 10.2196/66762 (PMC12271965; doi:10.2196/66762)

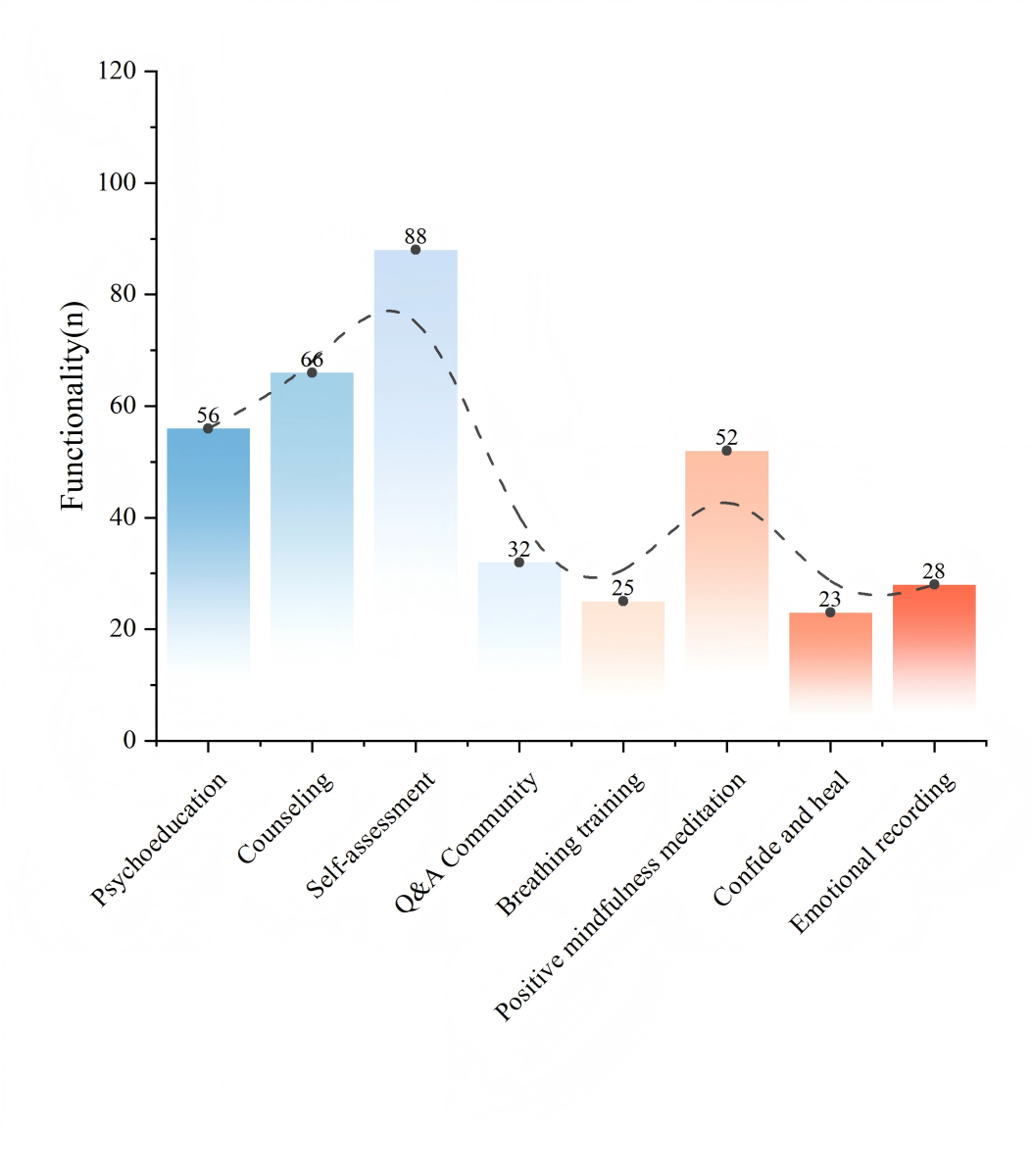

Supplement: Multimedia Appendix 2 [file jmir_v27i1e66762_app2.png]

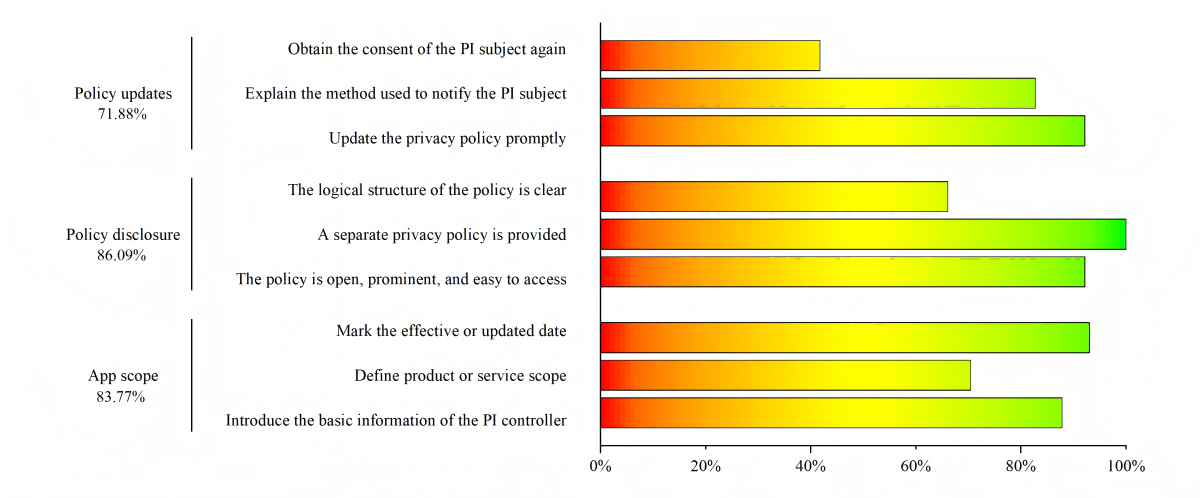

Supplement: Multimedia Appendix 4 [file jmir_v27i1e66762_app4.png]

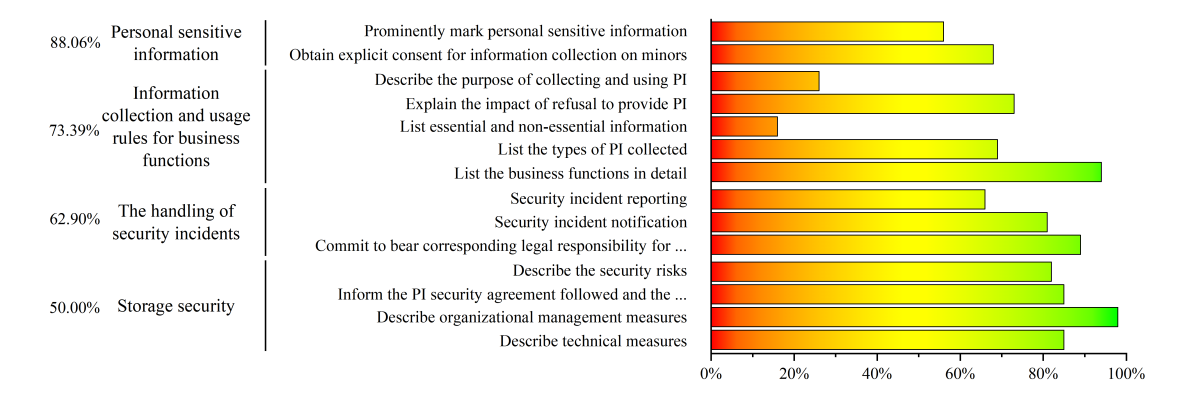

Supplement: Multimedia Appendix 5 [file jmir_v27i1e66762_app5.png]

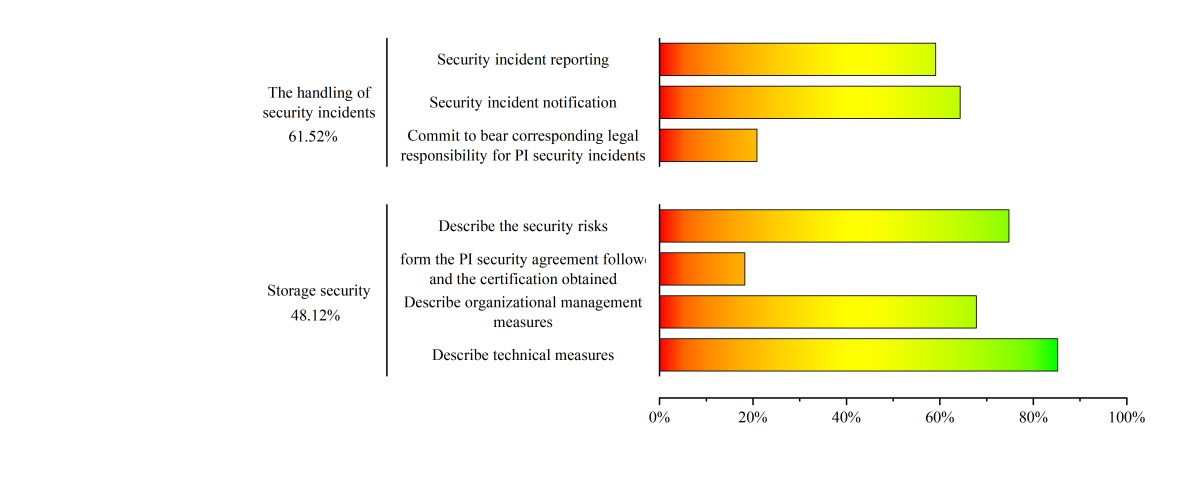

Supplement: Multimedia Appendix 6 [file jmir_v27i1e66762_app6.png]

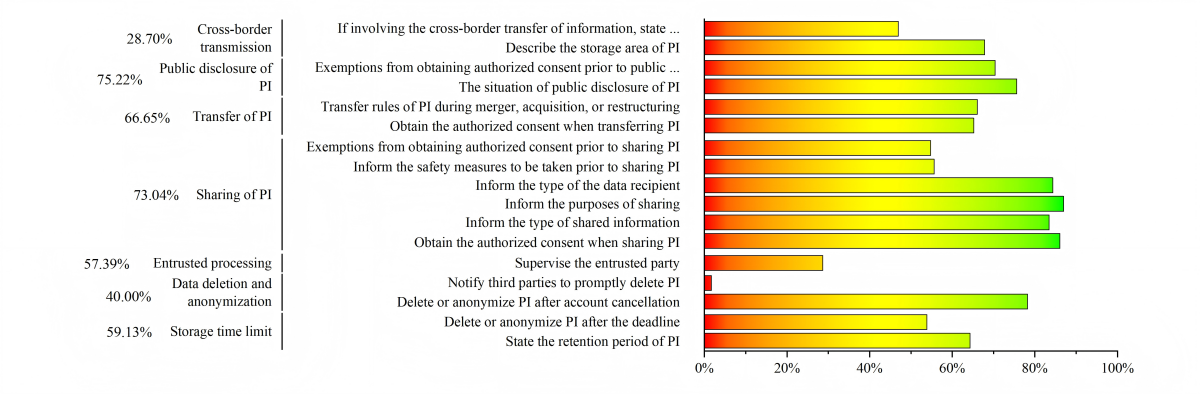

Supplement: Multimedia Appendix 7 [file jmir_v27i1e66762_app7.png]

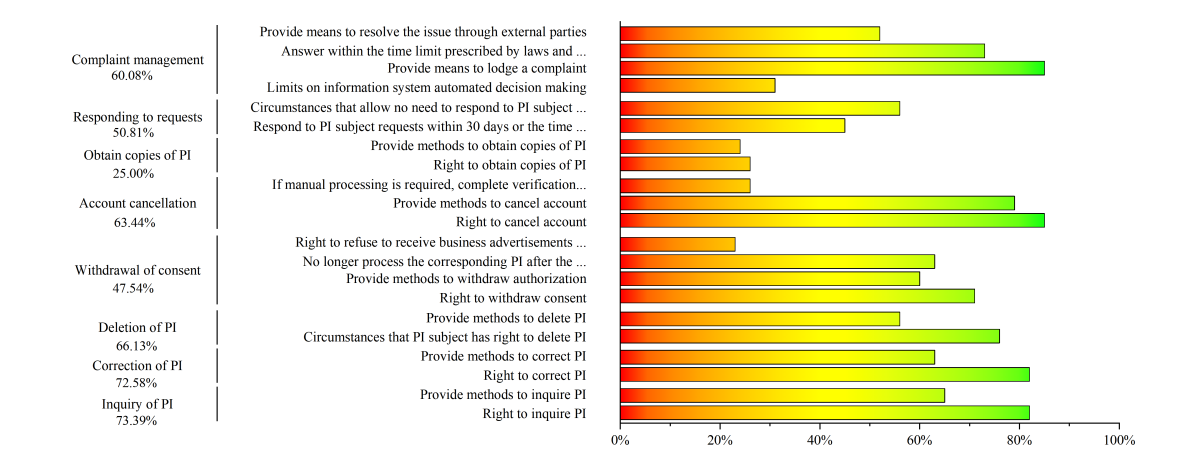

Supplement: Multimedia Appendix 8 [file jmir_v27i1e66762_app8.png]
